# Complete mitochondrial genome of the mulberry psyllid *Paurocephala sauteri* Enderlein (Hemiptera: Psyllidae) and phylogenetic analysis

**DOI:** 10.1080/23802359.2020.1846469

**Published:** 2021-01-11

**Authors:** Fu-ping Lu, Tao Geng, Hua-zhou Wu, De-zhao Lou, Na-Na Tu, Shu-chang Wang

**Affiliations:** a Environment and Plant Protection Institute, Chinese Academy of Tropical Agricultural Sciences, Haikou, China; b Hainan University, Haikou, China

**Keywords:** Mitogenome, *Paurocephala sauteri*, psyllid, phylogeny

## Abstract

*Paurocephala sauteri* (Enderlein, 1914) (Hemiptera: Psyllidae) is a species of a psyllid distributed in Asia. Mulberry is the only known host for *P. sauteri* until now. The complete mitogenome of *P. sauteri* (accession number: MT759765) 14,963 bp in size, including 13 protein-coding genes, 22 transfer RNAs, and two ribosomal RNAs genes. The base composition of the whole *P. sauteri* mitogenome is 40.26% for A, 7.86% for G, 34.07% for T, and 11.81% for C, with a high AT bias of 80.33%. The mitochondrial genome of *P. sauteri* was sequenced and annotated as the first representative of family Paurocephalidae. The present data could contribute to a detailed phylogeographic analysis of this valuable economic insect for further study in differentiating closely related species.

*Paurocephala sauteri* (Enderlein, 1914) (Hemiptera: Psyllidae) is a specie of psyllid distributed in China (Nantou Taiwan), Thailand, Philippines, India, Indonesia, Malaysia (Li 2011). Recent years, it was found more in mulberry planting area in Hainan province, China. Mulberry is the only known host for *P. sauteri* until now (Li 2011). Psylloids constitute a promising taxon for testing co-evolutionary hypotheses of insects and their host plants (Burckhardt and Basset 2000).The phylogenetic relationships within the monophyly of many groups of Psylloidea remains questionable, and one of these groups is the mainly tropical genus *Paurocephala*, which has been a continuing source of confusion (Mifsud and Burckhardt 2002). A lack of morphological synapomorphies makes the contribution of molecular data critical to reconstructing psyllid evolutionary history(Percy et al. 2018). *Paurocephala sauteri* belongs to the tropical genus of *Paurocephala*. Elucidating the sequence and structure of *P. sauteri* mitogenome is important for understanding its diversity and evolution.

Specimen of *P. sauteri* (Accession: PRJNA660576) were collected in Liangyuan Baodaoxincun, Danzhou, Hainan, China (109°49′ 66.88″E, 19°58′19.49″N) and deposited in the insect specimen room of Environment and Plant Protection Institute, Chinese Academy of Tropical Agricultural Sciences (Voucher number:LFP-SNS2020-1 (*P. sauteri*)), Haikou.

Genomic DNA of *P. sauteri* was extracted using insect gDNA isollation kit (Biomiga, China). Paired-end sequencing libraries with an insert size of ∼350 bp were constructed and the complete mitogenome were sequenced using Illumina Novaseq platform in Guangzhou Jierui Biotechnology Ltd., with a total data volume 10 G (150 bp, PE). High-quality reads were assembled from scratch using IDBA-UD and SPAdes (Gurevich et al. 2013). Protein-coding genes (PCGs) of the *P. sauteri* mitogenome were identified using BLAST search in NCBI, and tRNA genes were identified using the tRNAscan-SE search server (Schattner et al. 2005). The final assembled mitogenome was also verified on the MITOS web server (Bernt et al. 2013).

The complete mitogenome of *P. sauteri* (GenBank accession number MT759765) is 14,963 bp in size, including 13 protein-coding genes (*nad2*, *cox1*, *cox2*, *atp8*, *atp6*, *cox3*, *nad3*, *nad5*, *nad4*, *nad4l*, *nad6*, *cob*, *nad1*), 22 transfer RNAs (*trnI-ILE*, *trnQ-GLN*, *trnM-MET*, *trnW-TRP*, *trnC-CYS*, *trnY-TYR*, *trnL2*, *trnK-LYS*, *trnD-ASP*, *trnG-GLY*, *trnA-ALA*, *trnR-ARG*, *trnN-ASN*, *trnS1*, *trnE-GLU*, *trnF-PHE*, *trnH-HIS*, *trnT-THR*, *trnP-PRO*, *trnS2*, *trnL1*, *trnV-VAL*), two ribosomal RNAs genes (12S and 16S). The base composition of the whole *P. sauteri* mitogenome is 40.26% for A, 7.86% for G, 34.07% for T, and 11.81% for C, with a high AT bias of 80.33%.

Based on the concatenated 13 mitochondrial PCGs sequences of 16 species from Hemiptera, the maximum-likelihood method (Nei and Kumar 2000) was used to construct the phylogenetic relationship of *P. sauteri* with 15 other Hemiptera insects (Figure 1). The phylogenetic analysis was performed using MEGA7 software (Kumar et al. 2016). *Paurocephala sauteri* (Paurocephalidae: Paurocephala) was clustered with the *Diclidophlebia paucipunctata* (Liviidae: Diclidophlebia), which reasonably support for that “*Diclidophlebia* is one of the two possible sister genera of *Paurocephala*” (Mifsud and Burckhardt 2002). The phylogeography analyses presented here support for the traditional classification (Li 2011; Mifsud and Burckhardt 2002; Burckhardt 2018; Zendedel et al. 2016) and the molecular phylogenetic framework (Percy et al. 2018; Cho et al. 2019). Psyllids may exhibit high level of morphological divergence between relatively closely related species, often influenced by ecology (Percy et al. 2018). This mitogenome data might also be useful for guiding future classification and research, and in particular as a reference point for further evolutionary studies in psyllid.

**Figure 1.**
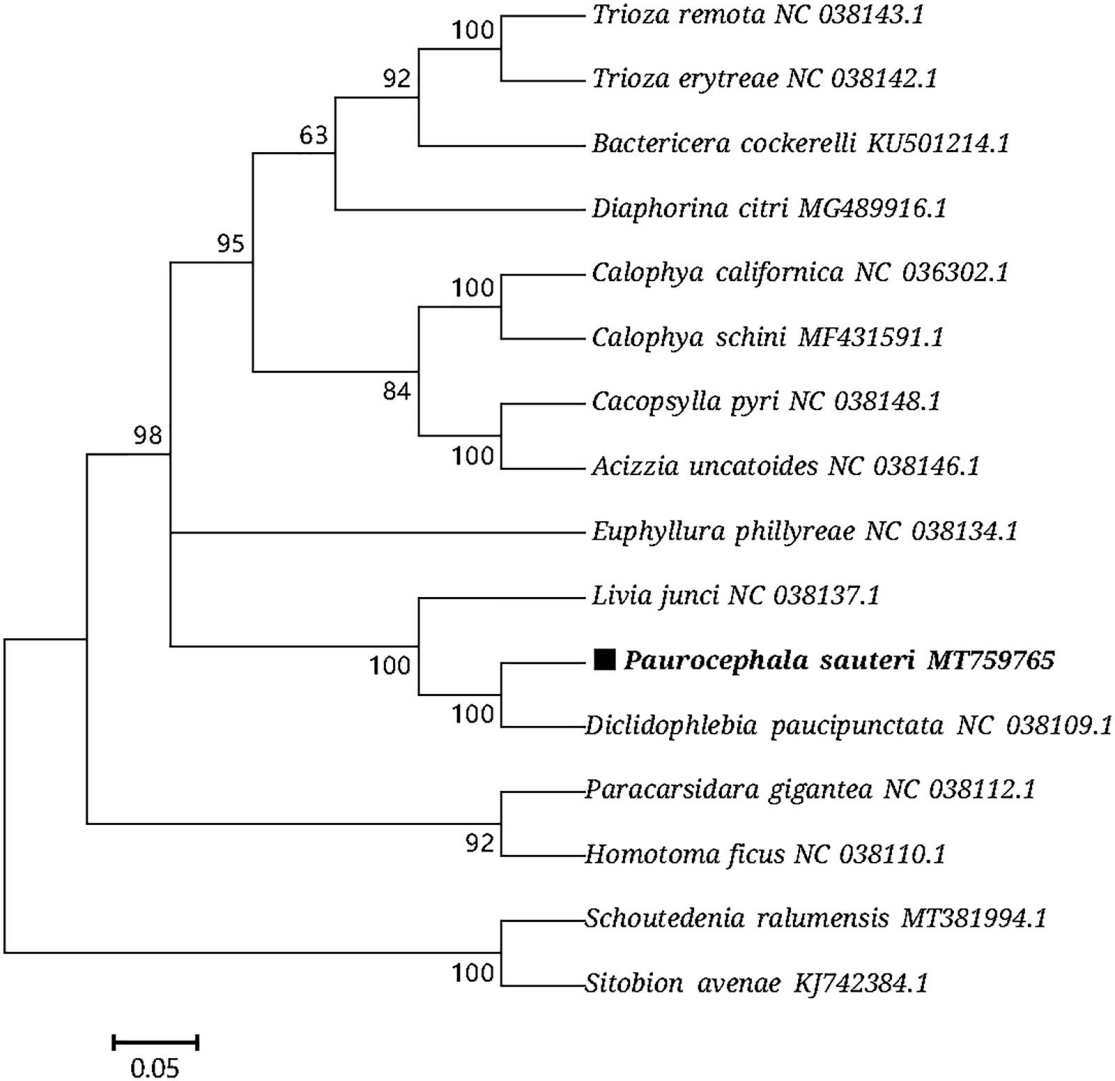
Phylogenetic tree showing the relationship between *P. sauteri* and 17 other Hemiptera insects based on maximum-likelihood method. Aphididae (*Schoutedenia ralumensis*) and Aphididae (*Sitobion avenae*) were used as outgroup. GenBank accession numbers of each sequence were listed in the tree with their corresponding species names.

## Data Availability

The genome sequence data that support the findings of this study are openly available in GenBank of NCBI at https://www.ncbi.nlm.nih.gov under the accession no. MT759765. The associated BioProject, SRA, and Bio-Sample numbers are PRJNA660576, SRX9048762 and SAMN15945249 respectively.References
